# Pre hospital administration of prothrombin complex concentrate in the head injured patient, a service evaluation

**DOI:** 10.1186/1757-7241-21-S1-S12

**Published:** 2013-05-28

**Authors:** RA Lendrum, J-P Kotze, AE Weaver

**Affiliations:** 1 London’s Air Ambulance, The Royal London Hospital, London, UK

## 

Prothrombin complex concentrate (PCC), provides rapid reversal of warfarin anticoagulation [1] and has been shown to reduce mortality and haematoma expansion secondary to non-traumatic intracerebral haemorrhage, in warfarinised patients [2]. The measurement of international normalised ratio (INR) and administration of PCC to warfarinised patients with an INR >2 and suspected intracranial haemorrhage, are interventions currently undertaken by London’s Air Ambulance (LAA). The feasibility of these interventions is illustrated by a case study of pre hospital administration of PCC and a service evaluation of their utilisation is presented.

## Methods

A retrospective database review was performed from the 1^st^ of July 2011 when PCC was introduced to the service, until the 31^st^ of October 2012.

## Case notes

Mrs C is an 84 year old female pedestrian, who was hit by a car doing approximately 30mph while crossing the road. On arrival of the aeromedical team, Glasgow Coma Score (GCS) was 10 (E3, V3, M4), the patient was discovered to have atrial fibrillation, be warfarinised (INR 2.8), with suspicion of intracranial haemorrhage. Rapid sequence induction of anaesthesia was performed and PCC administered during helicopter transfer. INR on arrival at the emergency department was 1.2. The patients CT scan showed evidence of temporal cerebral contusion and subarachnoid haemorrhage. Mrs C made a slow recovery and was discharged one month later.

## Results

2228 adult patients (>16 years) were attended during this period. 13 Successful INR tests were performed (<1% of all adult patients). Two warfarinised patients had an INR >2 with suspected intracranial haemorrhage and PCC (Octaplex) was administered successfully on both occasions.

## Discussion

The use of oral anticoagulants such as warfarin is continuously increasing. In warfarinised, head injured trauma patients, the incidence of intracranial haemorrhage and mortality rate are both increased [3]. The number of such patients attended by our service is low and only two patients to date have had reversal of INR with PCC. However, as this case illustrates, these interventions are suitable for pre hospital use and when delivered early after injury, have the potential to reduce haematoma expansion and potentially improve the outcome of this cohort of patients.
